# Flavor Chemical Research on Different Bee Pollen Varieties Using Fast E-Nose and E-Tongue Technology

**DOI:** 10.3390/foods13071022

**Published:** 2024-03-27

**Authors:** Chenshuo Liu, Enning Zhou, Yuying Zhu, Qiangqiang Li, Liming Wu

**Affiliations:** 1 Hainan Academy of Agricultural Sciences, Haikou 571100, China; 2 School of Food and Health, Beijing Technology and Business University, Beijing 100048, China; 3 Institute of Apicultural Research, Chinese Academy of Agricultural Sciences, Beijing 100093, China

**Keywords:** bee pollen, e-nose, e-tongue, flavor chemistry

## Abstract

Bee pollen, derived from various plant sources, is renowned for its nutritional and bioactive properties, aroma, and taste. This study examined the bee pollen with the highest yield in China obtained from four plant species, namely *Brassica campestris* (Bc), *Nelumbo nucifera* (Nn), *Camellia japonica* (Cj), and *Fagopyrum esculentum* (Fe), using fast e-nose and e-tongue technology to analyze their flavor chemistry. Results showed substantial differences in scent profiles among the varieties, with distinct odor compounds identified for each, including n-butanol, decanal, and ethanol, in Bc, Nn, and Cj, respectively. The primary odorants in Fe consist of E-2-hexen-1-ol and (Z)-3-hexen-1-ol. Additionally, e-tongue analysis revealed seven distinct tastes in bee pollen samples: AHS, PKS, CTS, NMS, CPS, ANS, and SCS, with variations in intensity across each taste. The study also found correlations between taste components and specific odor compounds, providing insights for enhancing product quality control in bee pollen processing.

## 1. Introduction

Bee pollen is formed via pollen collection by bees, which is then combined with saliva secretions and nectar. Due to its diverse health benefits, such as disease prevention, bee pollen has received heightened attention in food processing fields [1]. Bee pollen is rich in carbohydrates, essential amino acids, unsaturated fatty acids, vitamins, and various micronutrients [2]. It is a ‘complete food’ containing all amino acids necessary in the human diet [3]. Moreover, it demonstrates significant biological activities, including antioxidant, anti-inflammatory, and hypolipidemic effects [4,5]. Recent studies have also demonstrated the regulatory potential of bee pollen polysaccharides on intestinal microbiota [6]. In addition, recent research suggests that bee pollen may be used as a biofunctional ingredient for enhancing product quality, potentially incorporated into yogurt, cheese, bread, and fermented beverages. Bee pollen can be utilized as an additive in biomedical formulations for bioprinting, biopolymers, tissue engineering, and nanoparticle formulation [7]. Furthermore, the extensive number of plant origins and the diverse array of resulting flavors make it critical to conduct flavor chemistry investigations on bee pollen, underscoring its profound significance.

Electronic nose (e-nose) and electronic tongue (e-tongue) technologies are two essential branches of contemporary sensor technology, mimicking the olfactory and gustatory systems of humans to differentiate chemical constituents within samples. Using the overall characteristic response signal of a sample to simulate identification and conduct quantitative and qualitative analysis has been widely utilized across various domains due to its speed, ease of operation, and reproducibility. The utilization of e-tongue and e-nose in food quality control and production monitoring is prevalent. The applications of the e-tongue and e-nose, with prediction accuracies ranging from 80% to 96%, were significant in the field of food analysis [8]. The integration of diverse intelligent sensory algorithms has become ubiquitous, with particular emphasis placed on the incorporation of e-tongue and e-nose technologies. The HERACLES e-nose instrument utilizes rapid gas chromatography technology, significantly enhancing the efficiency of qualitative and quantitative analysis for complex odor samples. The ASTREE e-tongue system relies on the measurement of potential differences across sensors directly contacting liquids, allowing the assessment of taste variations among products and formulations. According to Xia et al., an e-nose can effectively collect information on aroma compounds using a sensor array, allowing the identification of changes in tea aroma during processing and determining the quality of tea [9]. Based on research by Estivi et al., the alkaloid content of lupin seeds debittered using different solvents and ultrasound for varying soaking times was determined, while the taste profile was assessed using e-tongue technology [10]. Banerjee et al. applied e-nose and e-tongue systems to evaluate black tea quality and determined that the integrated systems achieved higher classification accuracy relative to individual systems [11].

The utilization of e-nose and e-tongue technologies is immensely important for assessing bee pollen quality and flavor, due to their rapid and non-invasive characteristics. E-noses often consist of gas-sensitive sensors that selectively respond to volatile compounds found in different chemicals. Upon contact with these sensors, the conductivity of the volatile compounds from bee pollen undergoes changes, which identifies and differentiates odor components via signal processing and pattern recognition techniques. The e-tongue can be utilized to assess taste characteristics in bee pollen samples, potentially influencing the perception of their aroma. Consequently, the data obtained from the e-tongue can offer additional information to facilitate a more comprehensive understanding of the sensory properties of these samples.

Therefore, this study selected the bee pollen with the highest yield in China obtained from four plant species, including *Brassica campestris* (Bc), *Nelumbo nucifera* (Nn), *Camellia japonica* (Cj), and *Fagopyrum esculentum* (Fe), and conducted a flavor chemical research based on fast e-nose and e-tongue technology. Using e-nose and e-tongue systems for qualitative and quantitative analysis, we aimed to discern variations in odor profiles and taste profiles, respectively, across the varieties of bee pollen. This study aims to establish a solid scientific foundation for the processing and use of bee pollen while emphasizing the importance of quality control measures for its resulting products.

## 2. Materials and Methods

### 2.1. Sample Collection and Reagent Acquisition

The bee pollen samples were collected during respective flowering season of four plant species: *Brassica campestris* (Bc), *Nelumbo nucifera* (Nn), *Camellia japonica* (Cj), and *Fagopyrum esculentum* (Fe), cultivated at the apiary of the Institute of Apicultural Research of Chinese Academy of Agricultural Sciences (IAR, CAAS, Beijing, China). The collected samples underwent grinding and freeze-drying to obtain a powder, stored at −20 °C. Various n-alkane (nC6 to nC16) standards were purchased from ZZBIO Co., Ltd. (Shanghai, China) for GC analysis.

### 2.2. Scanning Electron Microscopy Examination on Bee Pollen Samples

Prior to performing scanning electron microscopy (SEM) analysis, bee pollen powders were dispersed in water and spread evenly onto tin foil. The samples underwent a drying process before mounting on metal stubs. A thin layer of gold was coated onto the samples before observation using a Hitachi S-750 SEM system manufactured by the Hitachi Company, Tokyo, Japan.

### 2.3. Preparation of Bee Pollen Samples for Analysis

A sample of 0.2 g of bee pollen powder was weighed into a headspace vial with a capacity of 20 mL, designed for e-noses. The vial was sealed using a PTFE liner and prepared as five parallel samples. Subsequently, the prepared samples were placed onto an automated sampler device to perform analysis utilizing the e-nose.

A sample of 5 g of bee pollen powder was dissolved in 100 mL of 40% ethanol via ultrasonic treatment. Subsequently, the prepared pollen solution was filtered using filter paper and carefully transferred into a specialized e-tongue beaker with a capacity of 25 mL. The e-tongue was allowed to measure the solution accurately.

### 2.4. E-Nose Analysis

Samples were analyzed using the HERACLES NEO ultra-rapid gas chromatography e-nose, following the experimental conditions outlined in Table 1. Data processing was performed using AlphaSoft 2023 software. Calibration was conducted employing a standard solution of n-alkanes (nC6 to nC16), and the retention times were converted to retention indices for qualitative analysis of compounds referring to the AroChemBase database.

### 2.5. E-Tongue Analysis

An e-tongue was used to identify taste indicators across diverse bee pollen samples. Prior to sample measurement, sensor activation, calibration, and diagnosis were performed to guarantee a consistent sensor status. The e-tongue system incorporated the 6th-generation sensor system, consisting of AHS, ANS, SCS, CTS, NMS, PKS, and CPS sensors alongside a standard reference electrode (Ag/AgCl), totaling seven sensors. Among them, AHS, ANS, SCS, CTS, and NMS exhibited sensitivity towards taste attributes of sourness, sweetness, bitterness, saltiness, and umami, respectively. PKS and CPS functioned as composite sensors [12]. To ensure precise detection, the experimental sample volume was 25 mL, while the sampling time was set at 120 s. It was observed that performing three repeated measurements yielded optimal testing conditions for data analysis.

### 2.6. Statistical Analysis

The statistical methodology employed in this study was consistent with the approach described in our previous publication [13]. A *t*-test was performed using SPSS version 21.0 (IBM Co., Armonk, NY, USA). The dataset utilized in the *t*-test followed a Gaussian distribution and exhibited homogeneity of variance, guaranteeing the validity of the test results. A significance level of 0.05 or lower was considered statistically significant, indicating a notable distinction between the two designated groups. For principal component analysis (PCA) and orthogonal partial least-squares discriminant analysis (OPLS-DA), we utilized SIMCA-P version 13.0 software (SSB Co., Svedala, Sweden).

## 3. Results and Discussion

### 3.1. Bee Pollen Morphology Analysis

**Figure 1:**
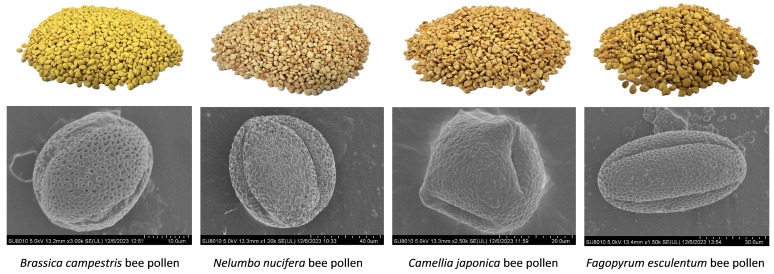
Physical and electron microscope images of four varieties of bee pollen.

A commonly utilized approach for determining the botanical origin of pollen loads is microscopic pollen analysis, as the size, shape, and surface properties of pollen grains are specific to particular plant species [14]. The micro-morphology of bee pollen is essential for its contribution to plant reproduction, and is linked to the pollination mechanism, genetic diversity, and adaptability of specific plants. The morphology, size, exine ornamentation type, and germination pore type are primary indicators for pollen examination using electron microscopy. As documented, pollen grains have intricate patterns along their outer walls, with the pollen coat seamlessly enveloping the outer wall layer and intricately sculpted surfaces [15,16]. Figure 1 illustrates electron microscopic images showcasing four distinct varieties of bee pollen. Upon examination of the electron microscope images, Bc exhibited pollen grains to be as follows: monad, radial, isopolar, tricolporate; reticulate, homobrochate, brochi coarse, lumina ca. 1.0 µm wide, muri very thin, simplicolumellate, columellae baculae shaped; colpus as long as grain, wide; polar shape circular; grains prolate to subprolate, ca. 30.0 µm long × 24.0 µm wide. The Nn sample exhibited pollen grains to be as follows: monad, radial, isopolar, tricolpate; verrucate, verrucae fine resembling small baculae; colpus ¾ as long as grain, thin; polar shape circular; grains subprolate, ca. 68.0 µm long × 60.0 µm wide. The Cj sample exhibited pollen grains to be as follows: monad, radial, isopolar, tricolporate; reticulate, brochi fine, muri simplicolumellate, baculae shaped; colpus as long as grain; pore inconspicuous, slightly protruding; polar shape triangular; grains suboblate, ca. 35.0 µm long × 36.0 μm wide. The Fe sample exhibited pollen grains to be as follows: monad, radial, isopolar, tricolporate; baculate, baculae coarse, lumina ca. 1.0 to 2.0 µm wide; colpus as long as grain, thin, ends acute; pore lalongate, depressed; grains subprolate, ca. 68.0 µm long × 36.0 µm wide. When examining the four varieties of bee pollen, there was a subtle disparity in hue, while most were predominantly yellow. The coloration of the Bc sample had enhanced vibrancy and luminosity, while the Fe sample’s color appeared comparatively deeper.

### 3.2. E-Nose Analysis of Bee Pollen

PCA operates as an unsupervised method to transform a set of possibly correlated variables into a linearly uncorrelated set of variables via orthogonal transformation. It is a valuable tool for data analysis and feature extraction that can be effectively combined with other pattern recognition algorithms to improve data separability and model performance [17]. PCA is a multidimensional data analysis approach with quantitative variables. Sample similarity represents small differences, and distance represents a noticeable component difference. PCA is typically employed to reveal the relationships among multiple variables via a few principal components or to extract a few principal components from the original variable while maintaining as much information about the original variable as possible [18]. PC1 is the predominant feature within the multidimensional data matrix, with PC2 following closely behind as the second most significant attribute in the dataset. PLS-DA is a supervised identification method predominantly used to identify the differences between samples of different classes. However, the model cannot identify variables and discard non-informational variables. OPLS-DA is an improved PLS-DA method using distinct projections and orthogonal components to characterize the variation between and within groups. OPLS-DA can eliminate data irrelevant to the category information (orthogonal) by orthogonalization. Additionally, compared to other approaches, it can more easily exclude independent variables unrelated to classification and screen out characteristic variables of samples. OPLS-DA is used to obtain optimal classification and establish discriminant models. OPLS-DA models have been widely utilized in food traceability or screening and the identification of differences in metabolomics [19]. Therefore, we utilized PCA and OPLS-DA to visually illustrate the distinctions in e-nose outcomes across four varieties of bee pollen.

**Figure 2:**
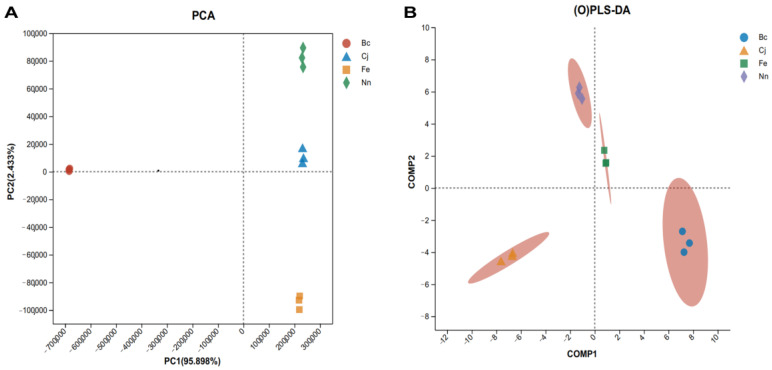
PCA score plot (**A**) and OPLS-DA score plot (**B**) of four varieties of bee pollen according to e-nose analysis.

The PCA score plot shown in Figure 2A illustrates the classification of four distinct varieties of bee pollen samples, with PC1 and PC2 accounting for a cumulative contribution rate of 98.331%. This encompasses the valid representation of the sample characteristics. The proximity of samples indicates their similarity, with closer distances reflecting smaller dissimilarities; conversely, greater separations between samples indicate more pronounced differences. The Bc sample is located independently on the left side of the designated area, while the remaining samples are positioned within the right-side region, indicating a significant disparity in overall olfactory characteristics between the Bc sample and its counterparts. There are discernible variations in the olfactory characteristics of the remaining three samples. Furthermore, the OPLS-DA score plot (Figure 2B) indicates distinct separation among the four pollen samples. Notably, the proximity between Nn and Fe in the score plot is comparatively closer than the other pollens, suggesting a lesser disparity between them relative to other pollen varieties. PCA and OPLS-DA revealed pronounced discrimination among the four varieties of bee pollen, indicating substantial variations in odor profiles across the samples.

**Figure 3:**
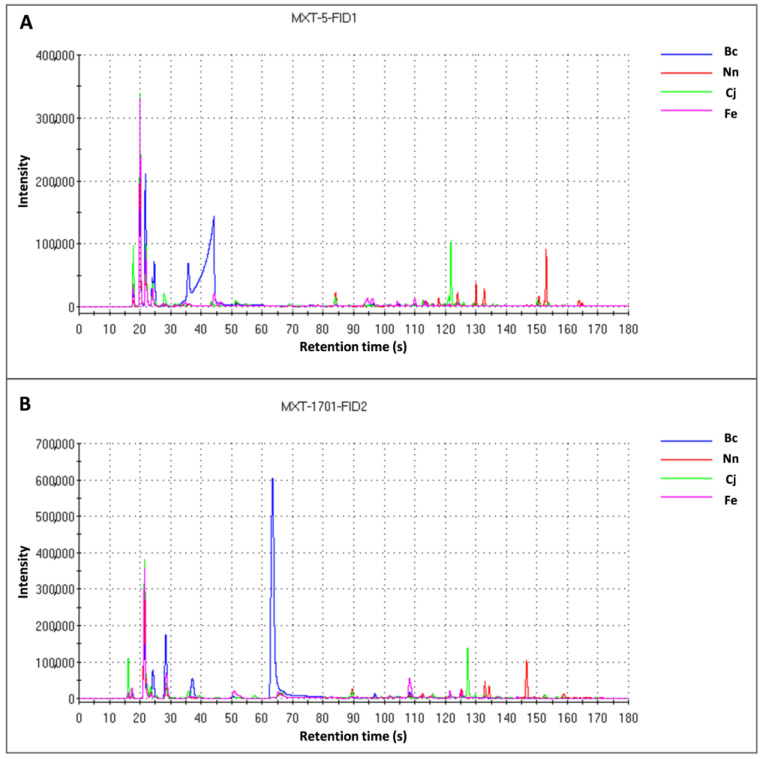
Gas chromatogram of the four varieties of bee pollen collected by MXT-5-FID1 (**A**) and MXT-1701-FID2 (**B**) columns, respectively.

The gas chromatography plots of distinct bee pollen samples (Figure 3) were produced with Origin version 2022 software. Based on the findings depicted in Figure 3, clear variations are present in the chromatographic data of different varieties of bee pollen. Initially, we utilized PCA and OPLS-DA to rapidly identify components in the samples that exhibit significant variations and contribute significantly to flavor. Subsequently, we employed the Arochembase database to determine volatile odor substances with specific characteristics. The detailed qualitative and quantitative findings are outlined in Table 2. Because the quantitative and qualitative compositions of volatile compounds are primarily associated with floral species and, to a lesser extent, with climatic conditions and geographical locations, each pollen type has a unique volatile compound profile [20]. For instance, the Bc sample contains a diverse range of compounds, including 2-methyl-2-propanol, propan-2-one, 2-propanol, n-butanol, and 2-methylbutanal. Significantly, the predominant presence of n-butanol contributes to the alcoholic, amyl alcohol, and banana-like aroma notes, as well as cheese-like undertones, with fermented and fruity characteristics identified in Bc reaching 740,380, which is approximately 100 times higher than that found in Nn (7689). 2-Methyl-2-propanol characterized the camphor odor of bee pollen from Bc reaching 123,605, which is six times more than that from Nn (19,961).

Additionally, the Nn sample exhibits significant levels of hexanal, myrcene, alpha-terpinene, benzeneacetaldehyde, 2-phenylethanol, camphor, methyl salicylate, decanal, beta-himachalene, and nonanoic acid hexyl ester. Notably, decanal and beta-himachalene are prominent contributors to the characteristic olfactory profile of Nn, encompassing aldehydic notes alongside smoky nuances and citrusy undertones. Fatty elements alongside floral and herbaceous hints reminiscent of lemon peel and orange zest are also present. The aroma also exhibits soapy aspects with subtle stew-like qualities while maintaining a sweet yet tallowy essence complemented by waxy undertones. In the Cj sample, significant levels of methyl formate, ethanol, 1-propanol, methyl propanoate, (E)-2-butenal, pent-1-en-3-ol, (E)-2-pentenal, linalool, and beta-caryophyllene were found. Among these compounds, ethanol dominates and contributes to the olfactory profile of Cj, characterized by alcoholic notes alongside fragrant and pleasant undertones accompanied by pungency and sweetness. The Fe sample exhibits substantial levels of E-2-hexen-1-ol, (Z)-3-hexen-1-ol, benzaldehyde, and amyl propanoate. Notably, E-2-hexen-1-ol and (Z)-3-hexen-1-ol, with relative contents reaching 24,072 and 21,817, dominate the odor profile of Fe, exceeding those in Nn by 30 and 20 times, respectively, characterized by notes of banana, cooked butter, freshness, fruitiness, leafiness, medicinal qualities, walnut-like nuances, earthy undertones with floral hints, and mossy accents along with an oily petal-like aroma.

Based on research conducted by Bi et al., certain volatile organic compounds, including styrene, limonene, nonanal, and hexanal, are pivotal constituents that contribute to the distinctive aroma of yellow bee pollen [21]. Because of the high protein and lipid levels in bee pollen, exposure to oxygen, heat, or enzymes triggers protein hydrolysis, fat decomposition, and enzyme oxidation. Consequently, these reactions enhance the Maillard reaction and Strecker degradation pathways, causing the formation of distinctive flavor compounds. According to research by Ni, a total of 147 volatile organic compounds were found in the Nn sample, with aldehydes and terpenoids comprising most of these compounds [22]. A total of 42 aldehyde compounds were found, with the highest concentration observed for 2-pental (E) in fresh Nn. Terpene compounds were the predominant volatile constituents in fresh Nn samples. According to research conducted by Cai et al., alcohols make up a significant proportion (69.27%) of the total volatile components found in bee pollen, while aldehydes, ketones, esters, phenolic acids, and sulfides collectively contribute to 9.6% of the overall volatile composition [23]. The concentration of 4.6-dimethyl-dodecane in Bc reached 13.03%. In bee pollen, a total of 40 characteristic aromatic components have been found, including trans-2-nonenic acid, nonanoic acid, 10-undecylenal, beta-cyclocitral, isopentenol, 5-hydroxymethylfural, linalyl acetate, ethyl nonanoate, geranyl propionate, and beta-caryophyllene. These compounds contribute to the development of a luscious creamy flavor, buttery fragrance, floral and fruity aroma, invigorating sensation, and a subtle hint of marine essence. Nakib et al. identified a total of 67 volatile compounds, classified into acids, alcohols, aldehydes, alkanes, aromatic alcohols, benzene derivatives, chromene derivatives, esters, furans, ketones, nitrile, nitrogen compounds, phenols, sulfur compounds, terpenes, and others [24].

### 3.3. E-Tongue Analysis of Bee Pollen

**Figure 4:**
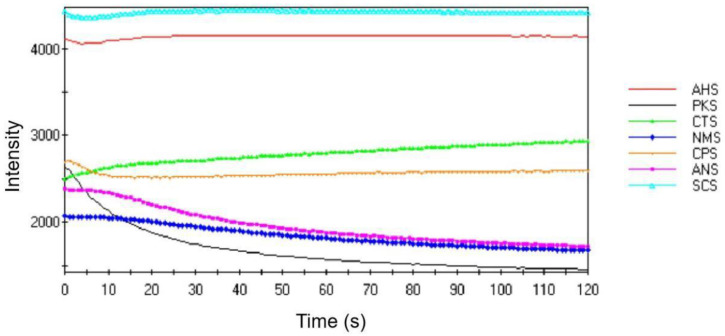
Original signal response graph of the *Brassica campestris* bee pollen (Bc) sample.

The response curve of an e-tongue is made up of three distinct stages: the baseline stage, the variation stage, and the stable stage. In our detection, the baseline phase was brief, whereas the change phase duration varied across sensors but typically reached stability within 30 s. The response curves of these seven taste sensors (AHS, PKS, CTS, NMS, CPS ANS, and SCS) can be categorized into three scenarios: (1) a progressive increase in the response signal over time; (2) a relatively stable response signal throughout the observation period; and (3) a gradual decline in the response signal over time. Taking Bc, for example (Figure 4), it notably exhibited a diverse complement of seven distinct tastes, each characterized by varying perceived intensities. Notably, AHS is the taste with the highest intensity, while PKS demonstrates a gradual decline in intensity over time. The results indicate that the e-tongue system has a remarkable capacity to elicit responses to the identified samples.

**Figure 5:**
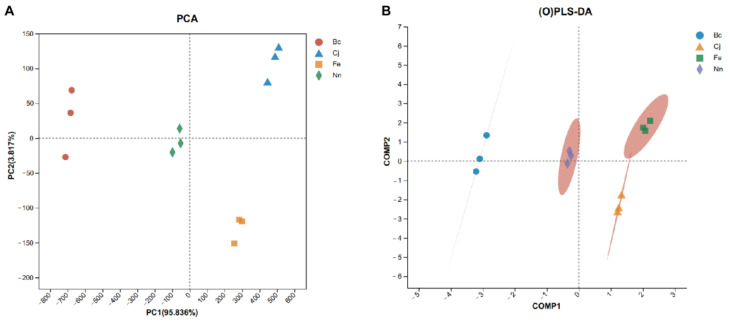
PCA score (**A**) and OPLS-DA score plots (**B**) of four varieties of bee pollen according to e-tongue analysis.

Moreover, notable variations in taste exist among different varieties of bee pollen samples. In Figure 5A, it is clear that both Bc and Nn are positioned towards the left side of the confidence interval, while Cj and Fe are situated on the right side. Additionally, there is a discernible disparity in the differentiating impact identified among these four pollen specimens. The cumulative contribution rates of PC1 and PC2 are 99.653%, effectively capturing the true representation of the samples. OPLS-DA analysis also indicated a significant distinction in taste profiles among the four types of bee pollen. The sample tastes exhibit a higher degree of similarity as the distance between them decreases. The higher proximity of Cj and Fe in the scoring chart relative to other pollen types implies a reduced divergence between them compared to other pollen.

**Figure 6:**
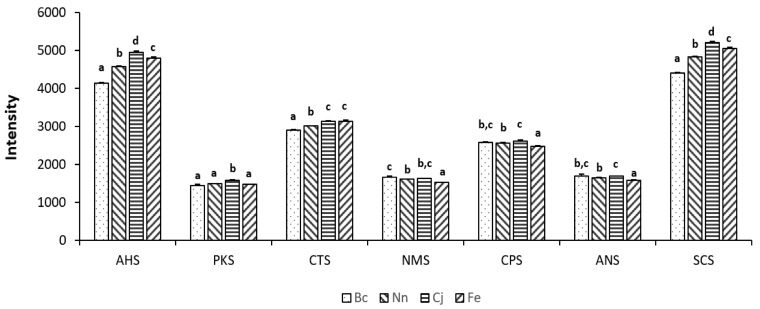
Bar chart illustrating the flavor differences between four varieties of bee pollen analyzed using an e-tongue. Different letter labels indicate significant differences.

A *t*-test was utilized to characterize the presence of a statistically significant distinction between the two datasets. The fundamental principle involves comparing the mean values of the two datasets, considering the extent of variability and sample size, to ascertain the presence of a statistically significant disparity between them. Based on the *t*-test analysis (Table 3 and Figure 6), notable differences were identified in the taste profiles among the four varieties of bee pollen. The trend of acidity (AHS) and bitterness (SCS) remained consistent and distinct across the four varieties, with the Cj sample exhibiting the most pronounced acidity and bitterness, while the Bc sample demonstrated the lowest expression of both. A noticeable disparity in saltiness (CTS) was observed between Bc and Nn, with the Bc samples exhibiting a comparatively milder saltiness profile, while no statistically significant differentiation was found between Cj and Fe samples. The umami (NMS) response exhibited significant differences between the Fe sample and the remaining three bee pollen varieties, with Fe demonstrating comparatively weaker umami taste. However, no statistically significant difference was observed between the Bc and Cj samples, as well as between the Nn and Cj samples. Notably, a discernible difference was present between the Bc and Nn samples. Regarding sweetness (ANS), significant differences were observed between the Fe samples and the other three varieties of bee pollen, with Fe exhibiting a comparatively lower sweet taste. No statistically significant difference was identified between Bc and Cj, as well as between Bc and Nn. However, there was a notable dissimilarity between Nn and Cj. Moreover, the intensity of AHS and SCS flavors in these four varieties of bee pollen was significantly higher compared to the intensity of other tastes.

**Figure 7:**
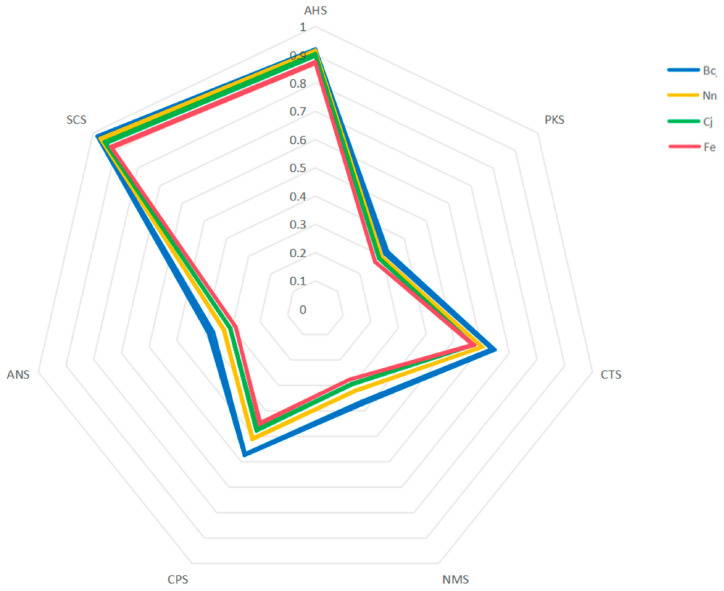
E-tongue radar chart for four varieties of bee pollen.

The radar chart presented in Figure 7 depicts the taste profiles of four distinct pollen samples, with the values representing the relative intensity of diverse tastes on a scale from 0 to 1. Discernible distinctions exist among the taste profiles of these four varieties of bee pollen. Moreover, this chart can be utilized to evaluate the relative strength of different tastes across various samples. The taste profile of SCS exhibits the highest relative intensity, while PKS has the lowest relative intensity. With respect to the taste intensity of SCS, ANS, CPS, and PKS, the four varieties of bee pollen can be arranged as Bc > Nn > Cj > Fe. The taste intensity of CTS remained consistent across both the Cj and Fe samples, while the Bc samples exhibited a higher intensity relative to the Nn samples. There was no significant variation in taste intensity for PKS and ANS across the four varieties of bee pollen. AHS has a slightly diminished taste profile compared to SCS in the four varieties of bee pollen.

### 3.4. Correlation Analysis between E-Nose and E-Tongue Datasets

**Figure 8:**
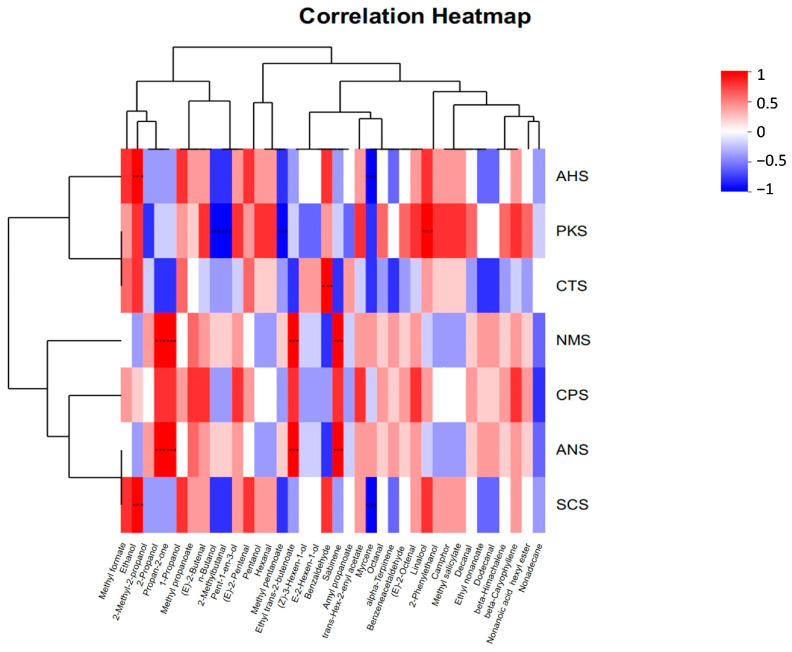
Correlation analysis between e-nose and e-tongue examination of bee pollen. *** *p*-value < 0.001.

Correlation analysis was utilized to assess the extent of correlation between two or more variables. As illustrated in Figure 8, each row corresponds to a distinct flavor, while each column represents an individual compound. The red hue signified a positive correlation between flavor and compounds, while a blue shade denoted a negative correlation. The intensity of color reflects the magnitude of this correlation. Our findings suggest that flavor components exhibited a positive correlation with the majority of esters, aldehydes, and alcohols, while underscoring a negative correlation with hydrocarbons. The flavor of PKS exhibited a positive correlation with linalool levels as well as a negative correlation with n-butanol, 2-methylbutanal, and ethyl trans-2-butenoate concentrations. The flavor of CTS has a positive correlation with benzaldehyde levels. The ANS and NMS levels positively correlate with propan-2-one, 2-propanol, methyl pentanoate and sabinene levels. The flavor of AHS and SCS exhibits a positive correlation with ethanol levels and a negative correlation with octanal concentrations. Furthermore, the correlation analysis findings for flavor components between ANS and NMS and AHS and SCS exhibited a substantial degree of similarity.

## 4. Conclusions

Flavor chemical analysis employing rapid e-nose and e-tongue techniques unveiled significant variations in both odor and taste among the pollen isolated from four distinct plant species, namely *Brassica campestris* (Bc), *Nelumbo nucifera* (Nn), *Camellia japonica* (Cj), and *Fagopyrum esculentum* (Fe). The analysis performed by the e-nose indicates that Bc, Nn, and Cj contain n-butanol, decanal, and ethanol as their primary odor compounds, respectively. In contrast, Fe predominantly consists of E-2-hexen-1-ol and (Z)-3-hexen-1-ol as its main odorants. Consequently, these substances exhibit distinct characteristics. The e-tongue analysis reveals that bee pollen samples offer a wide range of seven tastes: AHS, PKS, CTS, NMS, CPS, ANS, and SCS. Notably, there is significant variation in taste intensity across various bee pollen samples. By integrating the data acquired from both the e-nose and e-tongue analyses together, the taste components generally exhibited a positive correlation with esters, aldehydes, and alcohols while displaying a negative correlation with hydrocarbons. These findings serve as a theoretical foundation for the comprehensive processing and quality control of bee pollen products.

## Figures and Tables

**Table 1:** Heracles NEO instrument parameter configurations.

Parameters	Value
Headspace vial	20 mL
Sample amount	0.2 g
Incubation temperature	80 °C
Incubation time	20 min
Inlet volume	5000 μL
Inlet speed	125 μL/s
Inlet temperature	200 °C
Inlet duration	45 s
Initial trap temperature	40 °C
Split mode	10 mL/min
Injection duration	50 s
Final trap temperature	240 °C
Initial column temperature	40 °C (30 s)
RAMP	0.5 °C/s −60 °C (0 s) 2.0 °C/s −250 °C (15 s)
Acquisition time	180 s
Detector temperature	260 °C
FID	12

**Table 2:** Qualitative and relative quantitative results of gas chromatographic data of the four varieties of bee pollen samples.

Compounds	Retention Time -Column 5 (s)	Retention Time -Column 1701 (s)	CAS	Odor Description	Bc	Nn	Cj	Fe
Methyl formate	17.73	17.30	107-31-3	Agreeable; Fruity; Plum	11,469	4321	39,886	15,056
Ethanol	19.90	21.35	64-17-5	Alcoholic; Ethanol; Fragrant; Pleasant; Pungent; Sweet	141,776	165,650	256,029	212,202
2-Methyl-2-propanol	21.83	28.61	75-65-0	Camphor	123,605	19,961	30,873	51,927
Propan-2-one	24.01	23.46	67-64-1	Apple; Characteristic; Fruity; Pear; Solvent; Sweet; Violet	22,363	19,003	20,321	16,956
2-Propanol	24.79	24.32	67-63-0	Acetone; Alcoholic; Ethanol; Floral; Pleasant; Woody	40,371	1391	3655	0
1-Propanol	27.90	35.95	71-23-8	Alcoholic; Ethanol; Fermented; Fruity; Fusel; Plastic; Pungent	3874	2572	16,968	5417
Methyl propanoate	35.82	39.33	554-12-1	Apple; Fresh; Fruity; Rum; Strawberry; Sweet	3861	2370	10,038	3088
(E)-2-Butenal	43.42	57.49	123-73-9	Floral; Plastic; Pungent	1028	1046	8496	264
n-Butanol	44.30	63.51	71-36-3	Alcoholic; Amyl alcohol; Banana; Cheese; Fermented; Fruity; Fusel; Harsh; Medicinal; Oil; Sweet	740,380	7689	4109	26,918
2-Methylbutanal	46.37	51.96	96-17-3	Almond; Apple; Burnt; Cocoa; Coffee; Fermented; Fruity; Iodoform; Malty; Nutty; Sour	6561	850	811	6399
Pent-1-en-3-ol	51.37	66.32	616-25-1	Burnt; Butter; Fruity; Grassy; Horseradish; Meaty; Milky; Pungent; Vegetable	4994	9368	10,060	2565
(E)-2-Pentenal	69.10	80.49	1576-87-0	Apple; Fruity; Green; Oily; Orange; Pungent; Soapy; Strawberry; Tomato	1233	693	4055	2500
Pentanol	72.81	85.78	71-41-0	Alcoholic; Anise; Balsamic; Fruity; Fusel; Oil; Pungent; Sweet; Waxy	227	934	797	258
Hexanal	84.01	89.44	66-25-1	Acorn; Aldehydic; Fatty; Fishy; Fresh; Fruity; Grassy; Herbaceous; Leafy; Sharp; Sweaty; Tallowy; Vinous	2141	13,722	10,314	3073
Ethyl trans-2-butenoate	85.99	95.48	623-70-1	Alliaceous; Chemical; Pungent; Rum; Sweet	1260	602	596	846
Methyl pentanoate	88.45	90.92	624-24-8	Apple; Fruity; Nutty; Pineapple; Sweet	1844	1125	1563	700
E-2-Hexen-1-ol	94.74	102.21	928-95-0	Banana; Butter (cooked); Fresh; Fruity; Leafy; Medicinal; Walnut	2265	792	951	24,072
(Z)-3-Hexen-1-ol	96.11	98.92	928-97-2	Earthy; Floral; Fresh; Fruity; Leafy; Mossy; Oily; Petal	3033	999	1591	21,817
Benzaldehyde	104.01	121.48	100-52-7	Almond; Bitter; Bitter almond; Burnt sugar; Cherry; Fruity; Malty; Oil; Pepper; Sharp; Sweet; Woody	922	999	2759	4799
Sabinene	106.99	105.12	3387-41-5	Citrus; Fresh; Pepper; Pine; Spicy; Sweet; Turpentine; Woody	2592	2066	2318	1863
Amyl propanoate	109.21	112.56	624-54-4	Apricot; Fruity; Pineapple; Sweet	1036	968	989	8780
trans-Hex-2-enyl acetate	110.41	116.65	2497-18-9	Apple; Banana; Fresh; Sweet; Waxy	583	853	1075	0
Octanal	112.63	115.85	124-13-0	Aldehydic; Citrus; Fatty; Floral; Fruity; Lemon; Meat (boiled); Orange; Orange peel; Pungent; Soapy; Stew; Waxy	918	736	412	687
Myrcene	113.77	108.06	123-35-3	Balsamic; Fruity; Geranium; Lemon; Metallic; Plastic; Pleasant; Resinous; Soapy; Spicy; Sweet; Woody	6442	13,005	6597	5918
alpha-Terpinene	115.47	114.86	99-86-5	Citrus; Fruity; Gasoline; Lemon; Medicinal; Woody	2390	3955	2350	1503
Benzeneacetaldehyde	118.39	121.52	122-78-1	Cocoa; Floral; Grassy; Hawthorn; Honey; Hyacinth; Rose; Sweet	976	8042	1403	959
(E)-2-Octenal	119.98	122.99	2548-87-0	Burdock; Burnt; Fatty; Fruity; Mushroom; Nutty; Sour; Sweet; Tallowy; Waxy	2179	2319	2561	1538
Linalool	121.78	127.27	78-70-6	Anise; Bergamot; Citrus; Floral; Fragrant; Fresh; Fruity; Lavender; Lemon; Lily; Muscat; Oil; Parsley; Rose; Spicy; Sweet; Terpenic; Woody	1076	2472	47,271	1884
2-Phenylethanol	125.95	129.70	60-12-8	Floral; Flower; Honey; Lilac; Perfumery; Rose; Spicy	3241	14,188	11,858	4346
Camphor	129.99	132.94	76-22-2	Aromatic; Camphor; Fragrant; Leafy	1502	16,620	4886	1520
Methyl salicylate	132.73	134.34	119-36-8	Berry; Minty; Peppermint; Sweet; Winey; Wintergreen	464	12,074	866	586
Decanal	136.85	132.88	112-31-2	Aldehydic; Burnt; Citrus; Fatty; Floral; Herbaceous; Lemon; Orange; Orange peel; Soapy; Stew; Sweet; Tallowy; Waxy	2223	21,070	7094	1239
Ethyl nonanoate	139.29	137.78	123-29-5	Fruity; Rose; Rum; Waxy	1278	1283	1129	796
Dodecanal	146.23	144.73	112-54-9	Aldehydic; Caprylic; Citrus; Fatty; Floral; Herbaceous; Lily; Oily; Soapy; Waxy	783	2162	697	615
beta-Himachalene	150.69	152.62	1461-03-6	-	923	45,690	5719	459
beta-Caryophyllene	153.05	146.61	87-44-5	Fruity; Spicy; Sweet; Terpenic; Woody	254	1329	2735	207
Nonanoic acid hexyl ester	163.75	158.78	6561-39-3	Brandy; Floral; Fruity; Vegetable	274	11,476	789	246
Nonadecane	176.73	170.74	629-92-5	Alkane; Fuel; Fusel	58	391	25	122

**Table 3:** Significant differences in taste among four varieties of bee pollen, based on e-tongue analysis (*p*-value).

	Bc vs. Nn	Bc vs. Cj	Bc vs. Fe	Nn vs. Cj	Nn vs. Fe	Cj vs. Fe
AHS	0.001 *	0.000	0.001	0.000	0.000	0.000
PKS	0.099	0.021	0.149	0.003	0.012	0.002
CTS	0.020	0.005	0.000	0.000	0.016	0.988
NMS	0.047	0.150	0.008	0.069	0.000	0.003
CPS	0.365	0.216	0.044	0.013	0.003	0.001
ANS	0.142	0.757	0.032	0.023	0.001	0.002
SCS	0.001	0.001	0.001	0.000	0.000	0.000

* *p*-value < 0.05 indicates significant differences between each pair of bee pollen samples.

## Data Availability

The original contributions presented in the study are included in the article, further inquiries can be directed to the corresponding authors.

## References

[1] Thakur M., Nanda V. (2020). Composition and functionality of bee pollen: A review. Trends Food Sci. Technol..

[2] Giampieri F., Quiles J.L., Cianciosi D., Forbes-Hernández T.Y., Orantes-Bermejo F.J., Alvarez-Suarez J.M., Battino M. (2022). Bee products: An emblematic example of underutilized sources of bioactive compounds. J. Agric. Food Chem..

[3] Wang S., Bi Y., Zhou Z., Peng W., Tian W., Wang H., Fang X. (2022). Effects of pulsed vacuum drying temperature on drying kinetics, physicochemical properties and microstructure of bee pollen. LWT-Food Sci. Technol..

[4] Chen M., Yang X., Ji Z., Zhao H., Cheng N., Cao W. (2024). Combined treatment of drying, ethanol, and cold plasma for bee pollen: Effects on microbial inactivation and quality attributes. Food Biosci..

[5] Duan H., Dong Z., Li H., Li W., Shi S., Wang Q., Cao W., Fang X., Fang A., Zhai K. (2019). Quality evaluation of bee pollens by chromatographic fingerprint and simultaneous determination of its major bioactive components. Food Chem. Toxicol..

[6] Zhou W., Yan Y., Mi J., Zhang H., Lu L., Luo Q., Li X., Zeng X., Cao Y. (2018). Simulated digestion and fermentation in vitro by human gut microbiota of polysaccharides from bee collected pollen of Chinese wolfberry. J. Agric. Food Chem..

[7] Breda L.S., de Melo Nascimento J.E., Alves V., de Toledo V.D.A.A., de Lima V.A., Felsner M.L. (2024). Green and fast prediction of crude protein contents in bee pollen based on digital images combined with Random Forest algorithm. Food Res. Int..

[8] Lu L., Hu Z., Hu X., Li D., Tian S. (2022). Electronic tongue and electronic nose for food quality and safety. Food Res. Int..

[9] Xia H., Chen W., Hu D., Miao A., Qiao X., Qiu G., Liang J., Guo W., Ma C. (2024). Rapid discrimination of quality grade of black tea based on near-infrared spectroscopy (NIRS), electronic nose (E-nose) and data fusion. Food Chem..

[10] Estivi L., Buratti S., Fusi D., Benedetti S., Rodríguez G., Brandolini A., Hidalgo A. (2022). Alkaloid content and taste profile assessed by electronic tongue of *Lupinus albus* seeds debittered by different methods. J. Food Compos. Anal..

[11] Roy R.B., Tudu B., Shaw L., Jana A., Bhattacharyya N., Bandyopadhyay R. (2012). Instrumental testing of tea by combining the responses of electronic nose and tongue. J. Food Eng..

[12] Kim Y., Lee U., Eo H.J. (2023). Effect of NaCl pretreatment on the relationship between the color characteristics and taste of *Cirsium setidens* processed using a micro-oil-sprayed thermal air technique. Plants.

[13] Li Q., Sun M., Wan Z., Liang J., Betti M., Hrynets Y., Xue X., Wu L., Wang K. (2019). Bee pollen extracts modulate serum metabolism in lipopolysaccharide-induced acute lung injury mice with anti-inflammatory effects. J. Agric. Food Chem..

[14] Végh R., Csóka M., Sörös C., Sipos L. (2021). Food safety hazards of bee pollen—A review. Trends Food Sci. Technol..

[15] Dong J., Gao K., Wang K., Xu X., Zhang H. (2015). Cell wall disruption of rape bee pollen treated with combination of protamex hydrolysis and ultrasonication. Food Res. Int..

[16] Jabeen S., Zafar M., Ahmad M., Ali M.A., Elshikh M.S., Makhkamov T., Mamarakhimov O., Yuldashev A., Khaydarov K., Gafforov Y. (2024). Micrometer insights into *Nepeta* genus: Pollen micromorphology unveiled. Micron.

[17] Zhang Z., Zheng Z., He X., Liu K., Debliquy M., Zhou Y., Zhang C. (2024). Electronic nose based on metal oxide semiconductor sensors for medical diagnosis. Prog. Nat. Sci. Mater. Int..

[18] Huang G., Liu T., Mao X., Quan X., Sui S., Ma J., Sun L., Li H., Shao Q., Wang Y. (2023). Insights into the volatile flavor and quality profiles of loquat (*Eriobotrya japonica* Lindl.) during shelf-life via HS-GC-IMS, E-nose, and E-tongue. Food Chem. X.

[19] Kang C., Zhang Y., Zhang M., Qi J., Zhao W., Gu J., Guo W., Li Y. (2022). Screening of specific quantitative peptides of beef by LC–MS/MS coupled with OPLS-DA. Food Chem..

[20] Filannino P., Di Cagno R., Gambacorta G., Tlais A.Z.A., Cantatore V., Gobbetti M. (2021). Volatilome and bioaccessible phenolics profiles in lab-scale fermented bee pollen. Foods.

[21] Bi Y., Ni J., Xue X., Zhou Z., Tian W., Orsat V., Yan S., Peng W., Fang X. (2024). Effect of different drying methods on the amino acids, α-dicarbonyls and volatile compounds of rape bee pollen. Food Sci. Hum. Well..

[22] Ni J., Bi Y., Vidyarthi S.K., Xiao H., Han L., Wang J., Fang X. (2023). Non-thermal electrohydrodynamic (EHD) drying improved the volatile organic compounds of lotus bee pollen via HS-GC-IMS and HS-SPME-GC-MS. LWT-Food Sci. Technol..

[23] Cai Q. (2023). Analysis of Volatile Components and Identification of Main Aroma Components in Four Kinds of Bee Products.

[24] Nakib R., Rodríguez-Flores M.S., Escuredo O., Ouelhadj A., Coello M.C.S. (2022). Retama sphaerocarpa, Atractylis serratuloides and Eruca sativa honeys from Algeria: Pollen dominance and volatile profiling (HS-SPME/GC–MS). Microchem. J..

